# Nutritional analysis of rice landraces from southern Odisha, India

**DOI:** 10.1002/fsn3.3756

**Published:** 2023-10-13

**Authors:** Koustava Kumar Panda, Satpal Singh Bisht, Rojita Mishra, Parmeshwar Kumar Sahu, Amrita Kumari Panda, Roshan Subedi

**Affiliations:** ^1^ Department of Plant Biotechnology, M.S. Swaminathan School of Agriculture Centurion University of Technology and Management Paralakhemundi, Gajapati Odisha India; ^2^ Department of Zoology Kumaun University Nainital Uttarakhand India; ^3^ Department of Botany Polasara Science College Polasara, Ganjam Odisha India; ^4^ Department of Genetics and Plant Breeding Indira Gandhi Krishi Vishwavidyalaya Raipur Chhattisgarh India; ^5^ Department of Biotechnology Sant Gahira Guru University Ambikapur Chhattisgarh India; ^6^ Department of Life Sciences, School of Science Kathmandu University Dhulikhel Nepal

**Keywords:** environmental influence, landraces, nutritional profiling, phenotypic variation, proximate composition, rice

## Abstract

Rice landraces conserved by tribal farmers are important for their nutritional richness. Landraces are rich in essential amino acids, vitamins, anthocyanins, and flavonoids useful to cure noncommunicable diseases and metabolic disorders. A study was carried out with 10 rice landraces from the tribal‐dominated belt of Southern Odisha to investigate grain nutrition, proximate composition, and vitamin and mineral contents. The protein content of the landraces was higher (>6 g/100 g) and the fat content was lower (<0.6 g/100 g) than popular Indian rice varieties. The mean nutrient content of 10 rice landraces was as follows: protein 6.3 ± 0.313 g/100 g, total dietary fiber 1.6 ± 0.094 g/100 g, fat 0.536 ± 0.008 g/100 g, ash 10.514 ± 6.753%, and total sugar 77.18 ± 2.118 g/100 g. The high genotypic coefficient of variation (GCV) was observed for alkali spreading value (31.11%), capacity of grain hydration (52.705), index of hydration (171.439), moisture (46.343%), and vitamin B2 (23.994%) in rice landraces. Few landraces had superior iron content: Kalamalli (1.49 mg/100 g), Kandulakathi (1.42 mg/100 g), and Dudhamani (1.39 mg/100 g) compared to popular Indian rice varieties. Tikichudi had highest moisture (19%) and fat (0.53 g/100 g) content, which signifies the taste quality of rice. Kanakchudi exhibited the highest fiber content (1.8 g/100 g) and ash content (22.80%). Kalamalli contained higher zinc (0.49 mg/100 g), iron (1.49 mg/100 g), potassium (108.33 mg/100 g), magnesium (78.33 mg/100 g), and phosphorus (125.00 mg/100 g), whereas Muktabali was found to have higher Ca (3.88 mg/100 g) and Baunsidubraj exhibited higher niacin (4.9 mg/100 g). The indigenous landraces Kalamalli, Kandulakathi, and Dudhamani had considerably high iron content, whereas Kalamalli, Baunsidubraj, and Muktabali possessed less phytic acid in comparison with existing varieties and other landraces reported from various states of our country. Landraces Kalamalli, Kanakchudi, Tikichudi, and Muktabali from southern Odisha, India, represented nutritionally better genetic pool for future rice improvement.

## Graphical abstract

Rice landraces conserved by tribal farmers are important for their nutritional richness. In comparison with modern rice cultivars, landraces are rich in essential amino acids, various vitamins, anthocyanins, and flavonoids that can cure various noncommunicable diseases and metabolic disorders. The indigenous landraces Kalamalli, Kandulakathi, and Dudhamani had considerably high iron content whereas Kalamalli, Baunsidubraj, and Muktabali possessed lesser phytic acid in comparison with existing varieties and other landraces reported from various states of our country. Landraces Kalamalli, Kanakchudi, Tikichudi, and Muktabali from Southern Odisha, India, were found nutritionally better genetic pool for future rice improvement.

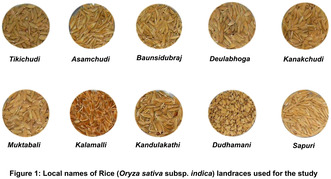

## 1 INTRODUCTION

Southern Odisha is considered a “Gene Treasury” for rice germplasm as the tribal communities (Bhottada, Gond, Paroja, Bhumia, Gadaba, Kandha, Langia Soura, Bondo, and Koya) domesticated a greater diversity of rice landraces with their traditional cultivation as well as conservation methods (Mishra, 2009). The rice landraces collected from the tribal villages of Southern Odisha were found to harbor many genes exhibiting resistance to diseases, insect pests, abiotic and biotic stresses, and rich in nutritional value, palatability, etc. (Tripathy et al., 2005). These landraces are the foundation of many allelic variations that are useful for breeding programs to develop new varieties with resistance characteristics against abiotic and biotic stresses (Longvah et al., 2020). The high diversity of landraces indicates better adaptation to changing climatic conditions.

Approximately 50,000 rice landraces exist in India (http://www.igmoris.nic.in, 2017). Odisha inhabits 10,000 rice landraces (Mishra et al., 2019). These landraces form an integral part of the sociocultural life of the tribal farmers who have been maintaining the genetic diversity of these races, but the advent of many high‐yielding rice varieties and modern agricultural practices have led to the loss of local germplasm and their genetic diversity (Roy et al., 2016). The nutritional and therapeutic values of landraces reported in ancient literature must be scientifically validated, as the phytocompounds and micronutrients from landraces, when consumed as food supplements, probably decrease the prevalence of noncommunicable diseases (Amudha et al., 2023).

Interest among consumers in having good‐quality nutritional rice with better micronutrients is increasing due to the rising incidence of nutritional disorders. Consumption of polished white rice over brown, unpolished indigenous varieties has been reducing the nutritional quality of rice. High calorie and low nutrient content have been one of the major problems for rice eaters in Asia. Rice breeders instantly need to develop better sustainable varieties with more nutritious grains and adaptation capabilities to changing agroecological conditions. There is inadequate information on the nutritional content of rice landraces in India (Lahkar et al., 2019; Longvah et al., 2020). Longvah et al. (2022) have already highlighted the importance of nutritional analysis of rice landraces for developing better varieties that can tolerate biotic and abiotic stresses. Therefore, the aim of the present study is the nutritional profiling of 10 ignored rice landraces from southern Odisha. The results of the study will generate information for molecular breeding programs of these exceptional rice varieties, and it can help local tribal farmers maintain their food security and socioeconomic status. Rice consumers is expected to benefit from the study as they get information regarding the nutraceutical value of these landraces.

## 2 MATERIALS AND METHODS

### 2.1 Seed collection

Seeds of all the rice landraces were collected from the Biju Patnaik Tribal Agro‐Biodiversity Centre, Jeypore, Odisha, which is a regional center of the M.S. Swaminathan Research Foundation (MSSRF). Farmers are conserving 50 folk rice varieties from Odisha, India, out of which a total of 10 rice landraces were selected for this study. The rice seeds were collected from notified villages under MSSRF across four districts of Southern Odisha during July–August 2018 (Figure S1). All these 10 landraces are being grown on MSSRF's farms, located in the districts of Phulbani, Koraput, Malkangiri, and Rayagada (Odisha, India) over the past 15 years. Four districts under southern Odisha, namely, Koraput, Rayagada, Nawarangpur, and Malkangiri, have been identified as the agrobiodiversity regions conserving the landraces of rice. The seed collection team, along with a scientist from the Biju Patnaik Tribal Agro‐Biodiversity Centre in Jeypore, surveyed the notified villages and collected the landraces. Genetic purity of each landrace was maintained by removing probabilities of cross‐pollination between other varieties grown on neighboring farms by using the flowering asynchrony method (Deb, 2006). During collection, the farmers were cross‐examined to acquire information about the landraces: the meaning of the landrace name, source or origin, grain quality, and yield.

### 2.2 Chemicals and reagents

Chemicals and reagents used in the research were of analytical grade and purchased from Fisher Scientific, Molychem, or Merck. HPLC‐grade solvents were used in the mobile phase of liquid chromatography.

### 2.3 Physical properties

The grain quality parameters were estimated by following the standard protocol according to the Directorate of Rice Research, Hyderabad, India (DRR, 2014).

#### 2.3.1 Grain length–breadth ratio

The length and breadth of grains were measured manually by using scales and graph sheets. Ten rice grains were selected randomly from the whole rice grains, and the L/B ratio was calculated by taking the mean value of 10 grains.

#### 2.3.2 Density of grain

One gram of grain was placed in a graduated test tube, and 0.1 mL of distilled water was added to it. The density was calculated by the differences between the initial value in the graduated test tube containing 1 mL of water and that of the test tube with 1 g of grain. The density was represented in g/cm^3^.

### 2.4 Functional properties

#### 2.4.1 Volume expansion ratio

The volume expansion ratio was calculated by dividing the volume of cooked rice by the volume of raw rice (Juliano, 1985). An equal volume of rice was cooked for each landrace.

#### 2.4.2 Elongation ratio

The elongation ratio was calculated by dividing the ratio of the length of cooked rice by the length of milled rice (Juliano, 1971). The length of 10 rice grains after cooking (intact at both ends) was measured using slide calipers, and the average length was determined. The elongation ratio was measured by dividing the average length of the cooked grain by the average length of the uncooked rice grain.

#### 2.4.3 Alkali spreading value

The alkali spreading value was estimated following the standard procedure (Juliano & Villareal, 1993). Six grains from each rice sample were soaked and spaced uniformly in a Petri dish containing 10 mL of a 1.7% potassium hydroxide solution. The Petri dish was roofed, undisturbed, and kept at a temperature of 30°C for 23 h in an incubator. The spreading of each grain was rated visually on a 7‐point numerical scale.

#### 2.4.4 Water absorption capacity

The water absorption ratio was calculated by dividing the weight of cooked rice to that of raw rice (Juliano, 1985).

#### 2.4.5 Capacity of grain hydration

The grains of different rice landraces were studied for their various physical characteristics such as capacity of grain hydration, index of hydration, capacity of inflation by grain, and energy value (Kouakou et al., 2008).

The capacity of grain hydration was determined by using the formula given below.

$$\mathrm{CHG}\;\left(g\right)=\frac{\text{Weight of soaking grains}-\text{Weight of grains before Soaking}\;}{\text{Number of grains}}$$

#### 2.4.6 Index of hydration

The index of hydration was determined using the following formula:

$$\mathrm{IH}=\frac{\text{Capacity of hydration}\;\mathrm{per}\;\text{grain}\;}{\text{Weight of}\;a\;\text{grains}}$$

#### 2.4.7 Capacity of inflation by grains

The capacity of inflation by grain (CIG) was then determined as follows.

$$\mathrm{CIG}\;\left(g\right)=\frac{\text{Volume after soaking}-V\text{olume before soaking}\;}{\text{Number of grains}}$$

#### 2.4.8 Amylose content

Ten milligrams of rice flour from each variety was placed in 100‐mL volumetric flasks, followed by the addition of 1 mL of 95% ethanol and 0.9 mL of NaOH. The contents were heated in a boiling water bath for 10 min and diluted to 100 mL. Five milliliters of sample solution, 1 mL of acetic acid, and 1.5 mL of iodine solution were added to a 100‐mL volumetric flask. The final volume was made to 100 mL with distilled water, and the suspension was mixed well and kept for 20 min. The samples were measured in two replicates at a wavelength of 620 nm (Perez & Juliano, 1978).

#### 2.4.9 Gel consistency

One hundred milligrams of rice flour was taken in a long test tube, and 0.2 mL of ethanol containing 0.25% thymol blue with 2.0 mL of KOH in distilled water was added and mixed well using a vortex mixer. The tubes were placed in a boiling water bath for 8 min, cooled for 5 min, mixed thoroughly, and kept in an ice bath for 20 min. The tubes were then removed and laid horizontally for 1 h, and measurements were made using graph paper. The degree of disintegration and the transparency of the paste dissolved out of the kernels were evaluated using a 7‐point scale (Bhattacharya, 1979). The gel consistency of each studied landrace was measured in two replicates.

#### 2.4.10 Phytic acid content

Phytic acid content was estimated using Wade reagent containing 0.03% FeCl_3_.6H_2_O + 0.3% sulpho‐salicylic acid (Gao et al., 2007). Phytic acid content of each landrace was measured in two replicates.

### 2.5 Proximate composition analysis

#### 2.5.1 Moisture content

Moisture content was estimated gravimetrically after uniformly drying the samples in a preheated oven (AACC, 2000). The moisture content in each rice flour sample was determined by placing 2–3 g of flour in dried tarred moisture cans and placing the containers in a well‐ventilated hot air oven maintained at a temp of 105 ± 5°C for 14–16 h. The covered cans were then weighed after cooling to room temperature in a desiccator to determine the loss in weight, and the value was calculated as a percentage of moisture loss (AACC, 2000 method No. 44‐15 A).

#### 2.5.2 Ash content

Ash content was determined gravimetrically after reducing the samples into inorganic matter in a muffle furnace (AACC, 2000). The ash content in each rice flour sample was estimated by placing samples in a muffle furnace (SIMECO) at a temperature of 550 ± 50°C till white gray residue was obtained (AACC, 2000 method No. 08‐01). Five‐gram flour samples were placed in clay crucibles and kept inside a muffle furnace at 550°C. They were incinerated until light gray ash or constant weight was obtained (7 h). After cooling, the samples were weighed, and the ash contents were calculated.

#### 2.5.3 Crude protein content

The crude protein was determined by nitrogen determination using Kjeldahl method (Kelplus Kelvac, model: KES12LR) (N X 5.95) conversion factor and the results were expressed as g/100 g according to Wang et al. (2016). About 1 mg of protein is digested with 500 mg of K_2_SO_4_ and 2.0 mL of CuSO_4_/H_2_SO_4_ Kjeldahl digestion solution. The digestion is initiated by maintaining the temperature at about 200°C until the fuming starts. The temperature is then raised until the acid boils gently at slightly above 400°C. The sample undergoes various color changes and finally becomes colorless, and the fuming ends. The digestion is continued further at this temperature for an additional 30 min. The total digestion time is 3–4 h. The temperature was controlled by fixed rheostat settings during digestion. A sample of 2.0 mL of water is also digested simultaneously to constitute the blank. Both sample and blank digests are made alkaline with NaOH and steam distilled. The ammonia liberated is absorbed in solutions of 2% boric acid and nitrogen contents are determined by titration with 10 mN HCl after blank correction.

#### 2.5.4 Total sugar content

The predried rice grain samples were ground to a fine powder using a mortar and pestle. The powdered rice samples were then immediately used for total sugar extraction. Extraction was made with both hot and cold water (Waite & Boyd, 1953). The extracts were estimated spectrophotometrically (LABINDIA^R^–ANALYTICAL UV 3000) using a phenol–sulfuric acid assay. The method is based on the absorbance of a colored aromatic complex formed between phenol and the carbohydrate at 490 nm.

#### 2.5.5 Crude fiber content

Crude fiber was quantified after chemical digestion of the test portion (AACC, 2000). The fiber content in the rice samples was determined by digesting the rice samples with 1.25% H_2_SO_4_ followed by digestion with 1.25% NaOH (AACC, 2000 method No. 32).

#### 2.5.6 Fat content

Crude fat content of the rice flour samples was determined in ether extract in Soxhlet apparatus (AACC, 2000). The crude fat content was determined in each rice flour sample by using petroleum ether as a solvent in a Soxhlet Extraction Heating Unit (solvent ether) (JSGW) according to the standard protocol (AACC, 2000 method No. 30‐10).

#### 2.5.7 Energy value in kilocalories

The energy‐yielding values such as protein, fat, and carbohydrates were multiplied as per Atwater's conversion factors (Merrill & Watt, 1973) to derive the total energy in terms of kcal/100 g of samples.

The energy value was obtained according to the following formula:

$$\mathrm{VE}=\left[9\times \text{Lipids}\;\left(\%\right)+4\times \text{proteins}\;\left(\%\right)+4\times \text{Carbohydrates}\;\left(\%\right)\right]\;\left(\text{AOAC},2003\right).$$

#### 2.5.8 Mineral analysis

The mineral contents in different rice flour samples were determined by digesting each rice variety in a di‐acid mixture consisting of HNO_3_:HClO_4_ on a hot plate (REMI Model: 2MLH) at a temperature of 180°C for 2 h. The digested samples were transferred to 100‐mL volumetric flasks, and the volume was made with double‐distilled deionized water and then filtered. The mineral contents, i.e., calcium, iron, and zinc in the digested samples, were then analyzed from the filtrate by means of an Atomic Absorption spectrophotometer (PerkinElmer) and an estimation of each element was carried out (AOAC, 2003).

#### 2.5.9 Vitamin B content analysis

Vitamin B1 content was analyzed by reversed‐phase high‐performance liquid chromatography using the C18 column (Hossain et al., 2019). Vitamin B2 and B3 were estimated by microbiological assay using *Lactobacillus casei*. Rice samples of 10 g were powdered using mortar and pestle and transferred into conical flasks, and 25 mL of the extraction solution was added and kept in a shaking water bath at 70°C for 40 min. Thereafter, the samples were cooled and filtered, and the final volume was made up to 50 mL with extraction solution. The sample was then filtered through 0.45 μm filter tips, and aliquots of 20 μL from the solution were injected into the HPLC by using an autosampler.

### 2.6 Statistical analysis

Statistical parameters were analyzed through AST v3.14 and OPSTAT software. Descriptive statistics and histograms were analyzed with the help of PAST v3.14 software. Analysis of variance (ANOVA) based on the RCBD was analyzed and then the relevant data were statistically analyzed for Duncan's multiple range test (DMRT) using the software package STATISTICA (StatSoft Inc., 1998). The genotypic coefficient of variation (GCV) and phenotypic coefficient of variation (PCV) were calculated. Principal component analysis (PCA) was performed through PAST v3.14 software by following the correlation‐based method. The proximal, functional, and nutritional characteristics of rice grains were estimated in two replications, data were averaged and presented in mean ± standard deviation.

## 3 RESULTS AND DISCUSSION

### 3.1 Physical properties

The bran colors of studied landraces were light brown (3), light yellow with the spine (1), brown (2), dark brown (2), speckled brown (1), and light yellow (1). Out of 10 landraces, eight had medium grain shapes, and the remaining two had bold and slender grain types (Table 1). The 100‐kernel weight was the maximum in *Kandulakathi* (2.9 g), followed by *Muktabali* (2.7 g), whereas *Dudhamani* had the lowest (1.3 g). A higher L/B ratio was observed for *Sapuri* (3.11), which was a slender‐shaped grain with the highest length (6.72 mm). Bagchi et al. (2016) reported the grain weight of a few rice landraces from West Bengal between the range of 1.56 g to 2.49 g and the highest length (7.99 mm) and breadth (2.19 mm). Rice with long grain sizes is mostly preferred during domestication and breeding programs. The grain dimensions such as length, width, and shape are important domestication traits in rice (Zhang et al., 2021).

**TABLE 1:** Physical properties of landraces from Southern Odisha, India.

Rice variety	Length (mm)	Breadth (mm)	L/B ratio	100 kernel Wt (g)	Density (g/cm^3^)	Color	Shape
*Tikichudi*	6.26^bc^ ± 0.028	2.34^cde^ ± 0.113	2.67^bc^ ± 0.007	2.5	1.25^e^ ± 0.007	Light brown	Medium
*Asamchudi*	6.68^a^ ± 0.042	2.22^cde^ ± 0.014	3.00^a^ ± 0.085	2.3	1.42^c^ ± 0.042	Light brown	Medium
*Baunsidubraj*	5.98^d^ ± 0.014	2.08^e^ ± 0.028	2.87^ab^ ± 0.028	2	1.66^a^ ± 0.014	Light yellow with spine	Medium
*Deulabhoga*	5.60^f^ ± 0.014	2.56^a^ ± 0.057	2.21^e^ ± 0.042	2.5	1.25^f^ ± 0.021	Brown	Medium
*Kanakchudi*	6.38^b^ ± 0.071	2.38^abc^ ± 0.042	2.68^bcd^ ± 0.156	2.8	1.25^ef^ ± 0.014	Brown	Medium
*Muktabali*	6.10^c^ ± 0.085	2.50^a^ ± 0.028	2.44^de^ ± 0.156	2.7	1.25^d^ ± 0.014	Light brown	Medium
*Kalamalli*	5.88^e^ ± 0.028	2.44^ab^ ± 0.028	2.40^bcd^ ± 0.283	2.3	1.66^b^ ± 0.028	Dark brown	Medium
*Kandulakathi*	6.26^bc^ ± 0.014	2.42^ab^ ± 0.042	2.58^bcd^ ± 0.028	2.9	1.25^f^ ± 0.021	Dark brown	Medium
*Dudhamani*	4.48^g^ ± 0.028	2.16^bcd^ ± 0.184	2.08^cde^ ± 0.014	1.3	1.25^e^ ± 0.007	Speckled brown	Bold
*Sapuri*	6.69^a^ ± 0.042	2.16^de^ ± 0.014	3.12^a^ ± 0.021	2.4	1.25^ef^ ± 0.006	Light yellow	Slender
Min	4.48	2.08	2.08	1.3	1.25	…..	….
Max	6.69	2.56	3.12	2.9	1.66	….	….
Mean	6.031	2.326	2.605	2.37	1.349	…..	…..
Std. error	0.203	0.051	0.106	0.145	0.054	…….	………
Stand. dev	0.641	0.162	0.334	0.46	0.172	……..	…..
Coeff. var	10.637	6.974	12.814	19.392	12.77	……….	…….

*Note*: Means followed by the same letter within rice landraces are not significantly different at 5% using Duncan's multiple range test (DMRT). Means of two replicates are taken.

### 3.2 Functional properties

The volume expansion ratio (VER) of rice landraces ranges from 1.10 to 1.22 and varied among the landraces, similarly ER ranges from 2.18 to 2.88. These findings indicate that *Tikichudi* and *Asamchudi* exhibit the highest elongation ratio, whereas *Kandulakathi* exhibited volume expansion rather than a high elongation ratio (Table 2). Consumers in Southeast Asia and industries prefer rice varieties that possess volume expansion due to quick satiety (Custodio et al., 2019). Alkali spreading value (ASV) of *Deulabhoga*, *Kanakchudi*, and *Dudhamani* had the lowest values (2.1–2.3), indicating that these varieties took much time to cook and have less water absorption capacity in comparison with another landrace, i.e., *Baunsidubraj* with highest ASV of 4.5 (Table 2). Amylose content (AC) varied among cultivars and showed the range of 1.10%–1.22%, and *Kalamalli* showed the highest AC, whereas *Deulabhoga* and *Sapuri* recorded the lowest AC values. The categorization of rice as per amylose content has been recommended by Juliano et al. (2012). The more the amylose content of rice, the harder the texture of cooked rice and vice versa for water absorption capacity and viscosity while amylose content also regulates starch digestion as starches with little amylose content digest easily in comparison with elevated amylose content (Bagchi et al., 2016).

**TABLE 2:** Functional properties of landraces from Southern Odisha, India.

Rice variety	VER	ER	ASV	WAC	CHG (g/grain)	IH	CIG (ml/grain)	AC (%)	GC (mm)	PA (%)
*Tikichudi*	1.12^f^ ± 0.014	2.88^a^ ± 0.028	4.1^b^ ± 0.021	2.10^ab^ ± 0.070	0.004^ab^ ± 0.003	0.190^a^ ± 0.001	0.019^ab^ ± 0.001	1.12^bc^ ± 0.014	2.86^a^ ± 0.014	6.7^cd^ ± 0.07
*Asamchudi*	1.19^c^ ± 0.007	2.88^a^ ± 0.014	4.2^b^ ± 0.141	1.98^c^ ± 0.544	0.002^b^ ± 0.004	0.017^b^ ± 0.001	0.017^ab^ ± 0.002	1.19^bc^ ± 0.007	2.87^a^ ± 0.014	6.85^cd^ ± 0.07
*Baunsidubraj*	1.17^d^ ± 0.007	2.50^c^ ± 0.071	4.5^a^ ± 0.021	2.41^a^ ± 0.049	0.001^b^ ± 0.001	0.012^c^ ± 0.001	0.012^bc^ ± 0.004	1.17^bc^ ± 0.014	2.50^d^ ± 0.099	6.5^cd^ ± 0.14
*Deulabhoga*	1.10^e^ ± 0.057	2.29^d^ ± 0.035	2.1^c^ ± 0.071	1.87^bc^ ± 0.004	0.003^ab^ ± 0.001	0.020^b^ ± 0.003	0.02^a^ ± 0.007	1.11^c^ ± 0.014	2.29^e^ ± 0.007	7.0^cd^ ± 0.14
*Kanakchudi*	1.15^e^ ± 0.007	2.73^b^ ± 0.035	2.3^c^ ± 0.071	1.77^bc^ ± 0.041	0.004^ab^ ± 0.001	0.021^b^ ± 0.001	0.021^ab^ ± 0.001	1.15^bc^ ± 0.007	2.73^c^ ± 0.007	7.05^bc^ ± 0.14
*Muktabali*	1.13^f^ ± 0.007	2.71^ab^ ± 0.120	4.3^b^ ± 0.071	2.10^ab^ ± 0.001	0.004^ab^ ± 0.001	0.021^b^ ± 0.001	0.021^ab^ ± 0.001	1.13^bc^ ± 0.021	2.71^c^ ± 0.021	6.55^bcd^ ± 0.74
*Kalamalli*	1.22^a^ ± 0.014	2.73^b^ ± 0.035	4.2^b^ ± 0.071	2.19^ab^ ± 0.001	0.003^b^ ± 0.001	0.016^bc^ ± 0.001	0.016^bc^ ± 0.003	1.22^ab^ ± 0.014	2.73^c^ ± 0.007	6.35^d^ ± 0.07
*Kandulakathi*	1.20^b^ ± 0.014	2.18^e^ ± 0.028	4.2^b^ ± 0.141	1.98^abc^ ± 0.002	0.005^a^ ± 0.001	0.021^b^ ± 0.001	0.021^ab^ ± 0.001	1.20^abc^ ± 0.014	2.19^f^ ± 0.004	6.80^cd^ ± 0.32
*Dudhamani*	1.20^b^ ± 0.014	2.81^a^ ± 0.014	2.2^c^ ± 0.141	2.01^abc^ ± 0.001	0.002^b^ ± 0.003	0.009^c^ ± 0.00	0.009^c^ ± 0.001	1.31^a^ ± 0.156	2.81^ab^ ± 0.014	9.65^a^ ± 0.00
*Sapuri*	1.10^g^ ± 0.014	2.80^ab^ ± 0.014	2.1^c^ ± 0.07	2.10^ab^ ± 0.008	0.005^a^ ± 0.00	0.002^d^ ± 0.001	0.022^ab^ ± 0.00	1.10^bc^ ± 0.028	2.80^bc^ ± 0.070	7.95^b^ ± 0.36
Min	1.1	2.18	2.1	1.774	0.001	0.002	0.009	1.1	2.19	6.35
Max	1.22	2.88	4.5	2.416	0.005	0.19	0.022	1.31	2.87	9.65
Mean	1.158	2.651	3.42	2.055	0.003	0.033	0.018	1.17	2.649	7.14
Std. error	0.014	0.078	0.341	0.056	0	0.018	0.001	0.02	0.076	0.312
Stand. dev	0.044	0.246	1.078	0.177	0.001	0.056	0.004	0.064	0.241	0.987
Coeff. var	3.814	9.268	31.516	8.613	40.53	168.847	24.388	5.436	9.08	13.817

*Note*: Means followed by the same letter within rice landraces are not significantly different at 5% using Duncan's multiple range test (DMRT). Means of two replicates are taken.

Abbreviations: AC, Amylose content; ASV, Alkali spreading value; CHG, Capacity of grain hydration; CIG, capacity of inflation by grain; ER, Elongation ratio; GC, Gel Consistency; IH, index of hydration; PA, Phytic acid; VER, Volume expansion ratio; WAC, Water absorption capacity.

Phytic acid (PA) is an antinutrient that reduces mineral bioavailability and inhibits calcium‐dependent starch‐digesting *α*‐amylase in the human gut. The larger the PA amount, the lesser the starch hydrolysis rate, which results in a lesser glycemic index (GI) value of food. The high PA content in rice affects starch digestibility, resulting in slower starch digestion and, consequently, low glycemic response (Kumar et al., 2020). PA content significantly inhibits calcium availability; as found in the present study, negative correlation was found between calcium and phytate (*r* = −.638, *p* < .01) (Table S1). Less phytate content in the studied landraces exhibits higher mineral bioavailability. There are reports that high phytic acid content is linked with poor bioavailability of minerals (Gupta et al., 2015). Phytate form complexes with minerals and make them inaccessible for absorption in the human intestine (Akter et al., 2020).

### 3.3 Proximate composition

The proximate composition of 10 rice landraces of Odisha showed that moisture content was found to be significantly varied between the varieties, where *Kanakchudi* had 6.00% as the lowest moisture content and the highest moisture contents were found for *Tikichudi* (19.0%). The highest ash content was observed for *Kanakchudi* (22.8%) and *Dudhamani* (22.8%) and the lowest was found for *Baunsidubraj* (4.65%). The quantity of ash present in any food sample plays a significant role in measuring the levels of essential minerals (Bhat & Sridhar, 2008). The total ash content of the studied rice varieties was found higher than that of rice varieties of Ethiopia (Tegegne et al., 2020).

Fat and protein content of all landraces showed that all studied varieties contain high protein content (>6 g/100 g) and low‐fat content (<0.6 g/100 g). These results are comparable with other reports that the Korean rice landraces contain 6.99 g/100 g protein (Li et al., 2014) and 6.7 g/100 g protein reported in LELLI AAM, Arunachal rice landraces (Longvah & Prasad, 2020) indicating the potentiality of these varieties as a functional food. On the contrary, Malhotra et al. (2022) studied nutritional profiling of rice landraces from northwest Indian Himalayas and found significant differences in protein content among rice landraces of Himachal Pradesh and Uttarakhand, for example, they reported protein 14.86 g/100 g in *Lamgudi* dhan which is much higher than the results of the present study. These variances are primarily associated with an alteration in their geographical origin (Kumar et al., 2015). The total crude fiber content of all studied landraces ranged between 1.5 and 1.8 g/ 100 g. The total sugar content of all varieties was higher than 75%; thus, all of them are considered good sources of carbohydrates (Table 3). *Muktabali* provided the highest energy among all studied varieties (361.26 kcal/100 g), followed by *Asamchudi* (342.68 kcal/100 g) and *Kanakchudi* (341.66 kcal/100 g), whereas the lowest value was found in *Dudhamani* (331.66 kcal/100 g).

**TABLE 3:** Proximate composition of landraces from Southern Odisha, India.

Rice variety	Moisture (%)	Ash (%)	Crude protein g/100 g	Total sugar g/100 g	Crude fiber g/100 g	Fat g/100 g	Food energy (kcal/100 g)
*Tikichudi*	19.0^a^ ± 0.70	9.05^c^ ± 0.21	6.50^b^ ± 0.071	76.3^d^ ± 0.283	1.5^cd^ ± 0.071	0.53 ± 0.07	335.97^cd^ ± 0.735
*Asamchudi*	6.50^c^ ± 0.21	5.50^f^ ± 0.085	6.50^b^ ± 0.141	78.0^bc^ ± 0.707	1.6^bcd^ ± 0.141	0.52 ± 0.11	342.68^b^ ± 0.410
*Baunsidubraj*	6.50^c^ ± 0.00	4.65^g^ ± 0.021	6.90^a^ ± 0.085	75.3^d^ ± 0.636	1.7^ab^ ± 0.071	0.53 ± 0.07	333.57^de^ ± 0.997
*Deulabhoga*	6.50^c^ ± 0.14	8.04^d^ ± 0.007	6.30^b^ ± 0.021	77.0^bd^ ± 0.707	1.6a^bcd^ ± 0.071	0.54 ± 0.21	338.06^c^ ± 0.728
*Kanakchudi*	6.00^c^ ± 0.29	22.8^a^ ± 0.42	6.20^b^ ± 0.113	78.0^b^ ± 0.707	1.8^a^ ± 0.070	0.54 ± 0.21	341.66^b^ ± 1.386
*Muktabali*	7.95^b^ ± 0.21	7.32^e^ ± 0.007	6.50^b^ ± 0.127	82.6^a^ ± 0.778	1.5^d^ ± 0.141	0.54 ± 0.20	361.26^a^ ± 0.651
*Kalamalli*	7.50^b^ ± 0.11	5.63^f^ ± 0.141	6.00^c^ ± 0.297	76.0^d^ ± 0.354	1.6^abcd^ ± 0.071	0.54 ± 0.01	332.86^de^ ± 0.481
*Kandulakathi*	7.25^b^ ± 0.43	8.05^d^ ± 0.014	5.80^c^ ± 0.212	76.0^d^ ± 1.909	1.5^bcd^ ± 0.014	0.53 ± 0.06	331.97^de^ ± 1.103
*Dudhamani*	6.50^c^ ± 0.07	22.80^a^ ± 0.354	6.10^bc^ ± 0.014	75.6^d^ ± 0.919	1.6^abc^ ± 0.141	0.54 ± 0.19	331.66^e^ ± 0.339
*Sapuri*	7.70^b^ ± 0.07	11.30^b^ ± 0.516	6.20^b^ ± 0.353	77.0^bd^ ± 0.707	1.6^bcd^ ± 0.141	0.55 ± 0.11	337.75^cde^ ± 3.528
Min	6	4.65	5.8	75.3	1.5	0.52	331.66
Max	19	22.8	6.9	82.6	1.8	0.55	361.26
Mean	8.14	10.514	6.3	77.18	1.6	0.536	338.744
Std. error	1.223	2.135	0.099	0.67	0.03	0.003	2.784
Stand. dev	3.869	6.753	0.313	2.118	0.094	0.008	8.804
Coeff. var	47.528	64.229	4.963	2.744	5.893	1.573	2.599

*Note*: Means followed by the same letter within rice landraces are not significantly different at 5% using Duncan's multiple range test (DMRT). Means of two replicates are taken.

### 3.4 Mineral and vitamin analysis

The mean iron content in studied landraces was 1.270 mg/100 g, with a total variability of 14% c.v. A similar mean iron concentration of 0.93 mg/100 g was also reported in 33 landraces of Arunachal Pradesh by Longvah and Prasad (2020). The maximum iron content of 1.49 mg/100 g was observed in *Kalamalli*, whereas few popular Indian rice varieties apparently contain relatively smaller iron content ranging from 0.85 to 1.45 mg/100 g in Swarna (MTU 7029), 1.03 mg/100 g in BPT 5204, and 1.18 to 1.2 mg/100 g in IR64 (Sellappan et al., 2009). However, the soil pH determines the micronutrients and the solubility of Fe decreases with each unit rise in soil pH (Abebe et al., 2017).

Commonly iron content tends to be elevated in red rice varieties compared to white rice varieties. The Fe concentration of rice landraces appears to have high geological changeability not only within India but also between countries, mainly due to edaphic and genetic factors. Kennedy and Burlingame (2003) reported that average contents of vitamin B1, B2, and B3 in brown rice were 0.457, 0.087, and 5.322 mg/100 g similar to the findings of the present study. Similar to the present findings, Longvah et al. (2022) reported the vitamin B1, B2, and B3 contents among rice landraces of Nagaland as 0.19–0.35, 0.04–0.07, and 2.48–3.77 mg/100 g, respectively. The study by Rafe and Sadeghian (2017) on Tarom and Domesiah rice cultivars from rice lands of Dargaz (Dargaz, Razavi Khorasan Province, Iran) and Kalat (Dargaz, Razavi Khorasan Province, Iran) reported Niacin (Vitamin B3) as the most abundant among studied B vitamins which is very much similar to our findings (Table 4).

**TABLE 4:** Mineral and vitamin B content of rice varieties mg/100 g.

Rice variety	Calcium	Iron	Zinc	Potassium	Magnesium	Phosphorous	B1	B2	B3
*Tikichudi*	3.63^ab^ ± 0.007	1.15^bcd^ ± 0.007	0.33 ± 0.04	101.33^c^ ± 0.54	65.00^d^ ± 0.424	117.33^e^ ± 0.226	0.44^ab^ ± 0.071	0.04^a^ ± 0.007	4.3^a^ ± 0.28
*Asamchudi*	3.51^ab^ ± 0.057	1.20^bc^ ± 0.028	0.28 ± 0.05	102.33^c^ ± 0.63	65.33^d^ ± 0.453	118.66^e^ ± 0.933	0.44^ab^ ± 0.049	0.05^a^ ± 0.014	4.3^a^ ± 0.21
*Baunsidubraj*	3.84^ab^ ± 0.134	1.06^d^ ± 0.014	0.46 ± 0.01	101.00^c^ ± 1.10	65.66^d^ ± 0.948	118.66^cde^ ± 0.403	0.5^a^ ± 0.00	0.06^a^ ± 0.014	4.9^a^ ± 0.14
*Deulabhoga*	3.06^ab^ ± 0.735	1.24^b^ ± 0.021	0.40 ± 0.54	101.00^c^ ± 1.16	66.33^d^ ± 0.714	123.66^a^ ± 0.544	0.39^b^ ± 0.007	0.05^a^ ± 0.007	4.6^a^ ± 0.07
*Kanakchudi*	3.13^bc^ ± 0.028	1.23^bc^ ± 0.021	0.39 ± 0.32	100.66^c^ ± 0.63	65.00^d^ ± 1.414	121.66^bc^ ± 0.721	0.41^ab^ ± 0.014	0.06^a^ ± 0.00	4.6^a^ ± 0.12
*Muktabali*	3.88^a^ ± 0.035	1.11^cd^ ± 0.007	0.42 ± 0.42	102.33^c^ ± 0.63	66.66^d^ ± 0.933	120.66^bcd^ ± 0.071	0.42^ab^ ± 0.007	0.06^a^ ± 0.007	4.5^a^ ± 0.07
*Kalamalli*	3.59^ab^ ± 0.021	1.49^a^ ± 0.106	0.49 ± 0.33	108.33^a^ ± 0.35	78.33^a^ ± 0.007	125.00^a^ ± 0.955	0.42^ab^ ± 0.099	0.06^a^ ± 0.495	4.6^a^ ± 0.21
*Kandulakathi*	3.35^abc^ ± 0.078	1.42^a^ ± 0.049	0.42 ± 0.01	104.00^b^ ± 0.70	77.00^b^ ± 0.707	120.66^ab^ ± 2.12	0.42^ab^ ± 0.021	0.06^a^ ± 0.014	4.5^a^ ± 0.21
*Dudhamani*	2.85^c^ ± 0.049	1.39^a^ ± 0.106	0.32 ± 0.29	102.66^c^ ± 0.75	68.66^c^ ± 0.148	118.33^de^ ± 0.629	0.43^ab^ ± 0.014	0.06^a^ ± 0.007	4.5^a^ ± 0.07
*Sapuri*	2.76^c^ ± 0.014	1.18^bc^ ± 0.028	0.39 ± 0.04	100.66^c^ ± 0.40	66.66^d^ ± 0.777	119.00^cde^ ± 0.714	0.43^ab^ ± 0.007	0.06^a^ ± 0.014	4.6^a^ ± 0.07
Min	2.76	1.06	0.28	100.66	65	117.33	0.39	0.04	4.3
Max	3.88	1.49	0.49	108.33	78.33	125	0.5	0.06	4.9
Mean	3.36	1.25	0.39	102.43	68.46	120.36	0.43	0.06	4.54
Std. error	0.13	0.04	0.02	0.74	1.57	0.78	0.01	0	0.05
Stand. dev	0.4	0.14	0.06	2.33	4.98	2.48	0.03	0.01	0.17
Coeff. var	11.79	11.33	16.53	2.28	7.27	2.06	6.67	12.49	3.77

*Note*: Means followed by the same letter within rice landraces are not significantly different at 5% using Duncan's multiple range test (DMRT). Means of two replicates are taken.

### 3.5 Variability parameters for various grain quality traits

The variability parameter showed that grain length varies from 4.480 mm (*Dudhamani*) to 6.690 mm (*Sapuri*), with an average of 5.99 mm. Rice breadth varies from 2.1 mm (*Baunsidubaraj*) to 2.52 mm (*Deulobhoga*), with an average of 2.325 mm (Table 1). The rice variety length/breadth ratio varied from 2.21 mm (*Deulobhoga*) to 3.125 mm (*Sapuri*) with an average of 2.63 mm. As per the standard evaluation system, IRRI (1996), three genotypes were short and bold type (*Dudhamani*, *Deulabhoga*, and *Kalamali*), one genotype was medium, slender type (*Baunsidubaraj*), and five varieties were long and bold type (*Tikichudi, Asamchudi, Kanakchudi, Muktabali*, and *Kandulakathi*). The gel consistency was lowest for Kandulakathi (2.19 mm) and highest for *Asamchudi (*2.87 mm), with an average of 2.631 mm. It is the criteria to determine the softness and hardness of rice after cooking. Amylose content determines the stickiness of rice after cooking. The amylose content of rice varieties ranges from 1.11% (*Deulobhoga*) to 1.31% (*Dudhamani*), with an average of 1.117% (Table 2).

A histogram describing the frequency distribution of various grain traits depicts that water absorption capacity (WAC), capacity of grain hydration (CG), and crude protein content displayed normal distribution (Figure S2 and S3). e  Skewness values for grain length (−1.715), breadth (−0.166), ER (−1.065), alkali spreading value (−0.450), WAC (−0.443), CIG (−0.806), gel consistency (−1.044), crude protein (−0.109), and calcium (−0.704) demonstrated that these traits were negatively skewed, whereas L/B ratio (0.385), density (1.334), VER (0.167), CHG (0.407), IH (3.075), amylose content (1.103), phytic acid (2.301), moisture (2.958), ash (1.432), total sugar (1.523), crude fiber (0.396), food energy (2.124), Fe (0.720), potassium (2.253), Mg (1.641), P (0.523), B1 (1.024), B2 (3.147), and B3 (0.712) were positively skewed. Positive skewness is linked with complementary gene interactions, while negative skewness is linked with duplicate gene interactions. There are reports that traits with skewed distribution are likely to be most dominant irrespective of whether they have a rising or falling effect on the trait (Sahu et al., 2017). In the present study, most traits showed skewness greater than 1 (Table S2), indicating a highly skewed distribution. Negative kurtosis values for breadth (−1.404), L/B ratio (−0.522), VER (−1.648), ER (−0.373), ASV (−2.192), CHG (−1.074), gel consistency (−0.415), crude fiber (−0.988), calcium (−0.689), Fe (−0.838), P (−0.975), and B3 (−0.728) indicate the absence of gene interactions and positive kurtosis values for length (3.812), density (0.038), WAC (0.452), IH (9.615), CIG (1.237), amylose content (1.134), phytic acid (5.917), moisture (9.061), ash (0.671), crude protein (0.811), total sugar (2.463), food energy (4.953), potassium (5.116), Mg (1.227), B1 (0.335), and B2 (9.930) (Table S2) indicate presence of gene interactions (Sahu et al., 2017).

### 3.6 Genetic variability parameters

Studies on coefficients of variation specified that the phenotypic coefficient of variation (PCV) was somewhat greater than the corresponding genotypic coefficient of variation (GCV) values for all studied 28 traits indicating that phenotypic variation was mostly determined by genotype with the insignificant influence of external factors (Sahu et al., 2017). The high genotypic coefficient of variation (GCV) observed for alkali spreading value (31.11%), CHG (52.705), IH (171.439), moisture (46.343%), and vitamin B2 (23.094%) indicates the possibility of selection of these traits with considerable improvement (Table S3). Similarly, Sahu et al. (2017) reported that GCV, along with heritability, determines the heritable variation.

### 3.7 Correlation analysis

Genotypic and phenotypic correlation coefficients among all the studied 28 traits were greater than their analogous phenotypic correlation coefficients showing the inherent resilient relationship among all the studied traits (Table S1). Grain length was correlated positively and significantly with the capacity of grain hydration (CHG), the capacity of inflation by grain (CIG), and vitamin B3 at the genotypic level. Water absorption capacities (WAC) were correlated positively and significantly with vitamin B contents. A significant positive correlation between total sugar and food energy was observed (*r* = 1.002**) at 0.01 level. Negative correlations between fiber and energy (*r* = −.465*) indicated that rice varieties with high food energy are low in fiber contents. A similar kind of correlation was observed with rice landraces in earlier reports (Lahkar et al., 2019). A positive correlation between ash and fiber (*r* = .602**) indicates that rice varieties with high fiber are likely to be high in ash content. The amount of ash present in food samples plays an important role in determining the levels of essential minerals (Bhat & Sridhar, 2008).

### 3.8 ANOVA analysis

Genetic variability is a prime factor for the survival of a species. Variations are the tools that can be explored for breeding programs. Analysis of variance (ANOVA) reported highly significant differences among most of the studied traits, but they are nonsignificant for the capacity of grain hydration, index of hydration, and vitamin B contents (Table S4). It indicates high variability among landraces (Manju et al., 2021). Similar to the present investigation, significant differences were also found among various traits such as protein (6.2–12.3), total dietary fiber (2.8–8.7), and fat (0.6%–3.2%) among rice landraces of Tamil Nadu, India (Ramadoss et al., 2023).

### 3.9 PCA analysis

**FIGURE 1:**
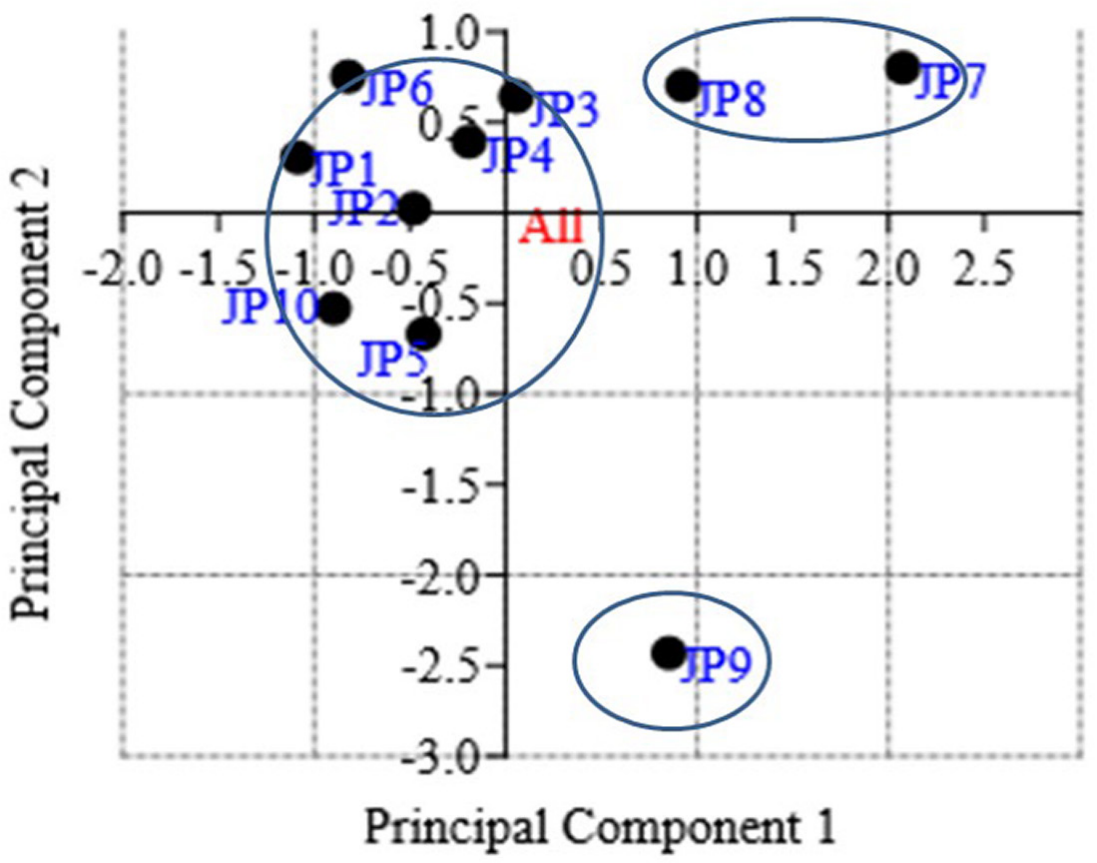
PCA scattered diagram.

**FIGURE 2:**
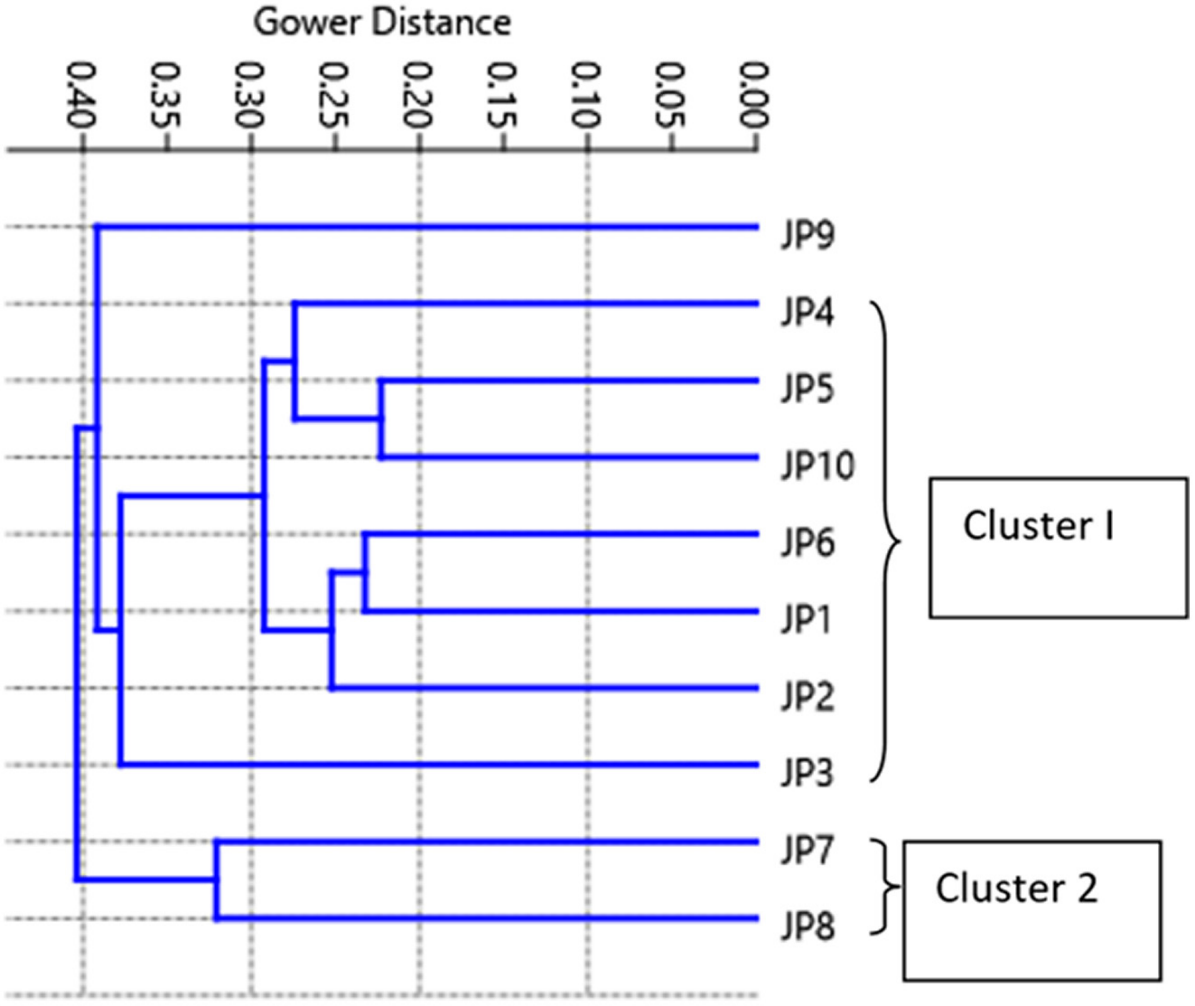
Cluster diagram for 10 rice landraces. JP9 genotype is highly diverse. JP1, JP2, JP3, JP4, JP5, JP6, and JP10 belong to cluster 1 and JP7 and JP 8 represent a separate cluster 2.

The principal component analysis (PCA) revealed six major variables, which accounted for 88% of the total variation from all the studied landraces. The principal component (PC1) contributed about 26.55% with an eigenvalue 7.43, PC2 contributed about 17.83% with an eigenvalue 4.99, PC3 contributed about 16.05% with eigenvalue 4.49, PC4 contributed about 12.48% with eigenvalue 3.49, PC5 contributed about 9.16% with eigenvalue 2.56, and PC6 contributed about 6.90% with eigenvalue 1.93 (Table S5). The rest of the PCs had less than 5% variability. The analysis detected that *Tikichudi* (JP1), *Muktabali* (JP6), *Kalamalli* (JP7), *Kandulakathi* (JP8), *Dudhamani* (JP9), and *Sapuri* (JP10) mainly contributed to PC1, whereas *Baunsidubraj* (JP3), *Kanakchudi* (JP5), JP6, JP7, JP8, JP9, and JP10 were the main contributing genotypes for PC2. It was postulated that about 44% of the total variation is represented by JP1, JP3, JP5, JP6, JP7, JP8, JP9, and JP10. A scattered diagram based on PCA (Figure 1) has grouped the landraces into four groups across PC1 and PC2. Cluster I comprised seven genotypes (70%). The scattered diagram showed that the genotype JP9 is highly diverse from the rest of the genotypes. A similar trend of PCA and variability analysis was found as observed in the scattered diagram in the present study. According to the PCA scatter diagram, the landraces, viz., *Kalamalli* (JP7) and *Kandulakathi* (JP8) belong to one cluster having high Zn and low phytic acid are distantly related with *Dudhamani* (JP9) (Figure 1) which possess low Zn and high phytic acid content. Further, landraces *Kalamali* (JP7) and *Dudhamani* (JP9) are the maximum contributing genotypes for PC1 and PC2 (Table S6) indicating their genetically diverse nature. There are reports that countless genetic variation exists in grain mineral contents of rice landraces (Dikshit et al., 2016). This study also reported that choosing genotypes with greater efficiency of Zn and Fe endospermic accumulation and their bioavailability may be an effective and reliable method to carry micronutrition benefits to the local population. Thus, crossbreeding among them could be valuable for the development of better genotypes with greater bioavailable Zn content. These crossbreed rice varieties will be more useful to improve Zn deficiency in humans as reported (Wang et al., 2021). These results are in agreement with the results of cluster analysis (Figure 2).

## 4 CONCLUSION

This is one of the pioneer works on the nutritional profiling and genetic variability of grain quality characters of rice landraces from southern Odisha, India. Proximate composition analysis results showed that *Kanakchudi* exhibited high fiber (0.85%) and ash contents (22.8%), *Baunsidubraj* revealed high protein (6.9 g/100 g), and *Muktabali* showed high sugar content (82.66.9 g/100 g) and food energy (361.26 Kcal per 100 g). The iron content of studied landraces is analogous to that of hybrid varieties. *Kalamalli* was found to be a nutritionally valued landrace as it contains the highest levels of Zn, Fe, K, Mg, and P. The vitamin B content of these landraces in southern Odisha was reported for the first time here and found to be equivalent to that of many improved varieties (e.g., genetically modified rice, Iksan 483 and Milyang 204, as reported by Choi et al., 2012). The low phytic acid content also indicates the vast dietary relevance of these landraces. The phenotypic coefficient of variation was greater than the parallel genotypic coefficient of variation for all studied 28‐grain quality traits, indicating that the phenotypic variation was determined only by genotype with the insignificant influence of environment, which is a good indicator for the selection of these traits for rice breeding programs.

## AUTHOR CONTRIBUTIONS

**Koustava Kumar Panda:** Conceptualization (lead); writing – original draft (lead). **Satpal Singh Bisht:** Conceptualization (supporting); supervision (supporting). **Rojita Mishra:** Writing – review and editing (equal). **Parmeshwar Kumar Sahu:** Data curation (equal); formal analysis (equal). **Amrita Kumari Panda:** Writing – review and editing (equal). **Roshan Subedi:** Writing – review and editing (supporting).

## FUNDING INFORMATION

This research received no specific grant from any funding agency.

## CONFLICT OF INTEREST STATEMENT

The authors declare that they have no known competing financial interests or personal relationships that could have appeared to influence the work reported in this paper.

## Supporting information


Figure S1

Figure S2

Figure S3

Table S1

Table S2

Table S3

Table S4

Table S5

Table S6

## Data Availability

The authors confirm that the data supporting the findings of this study are available within the article.
