# Supplementary figures and images for: Nutritional analysis of rice landraces from southern Odisha, India
Source: Food Sci Nutr. 2023 Oct 13;12(1):227–38. doi: 10.1002/fsn3.3756 (PMC10804119; doi:10.1002/fsn3.3756)

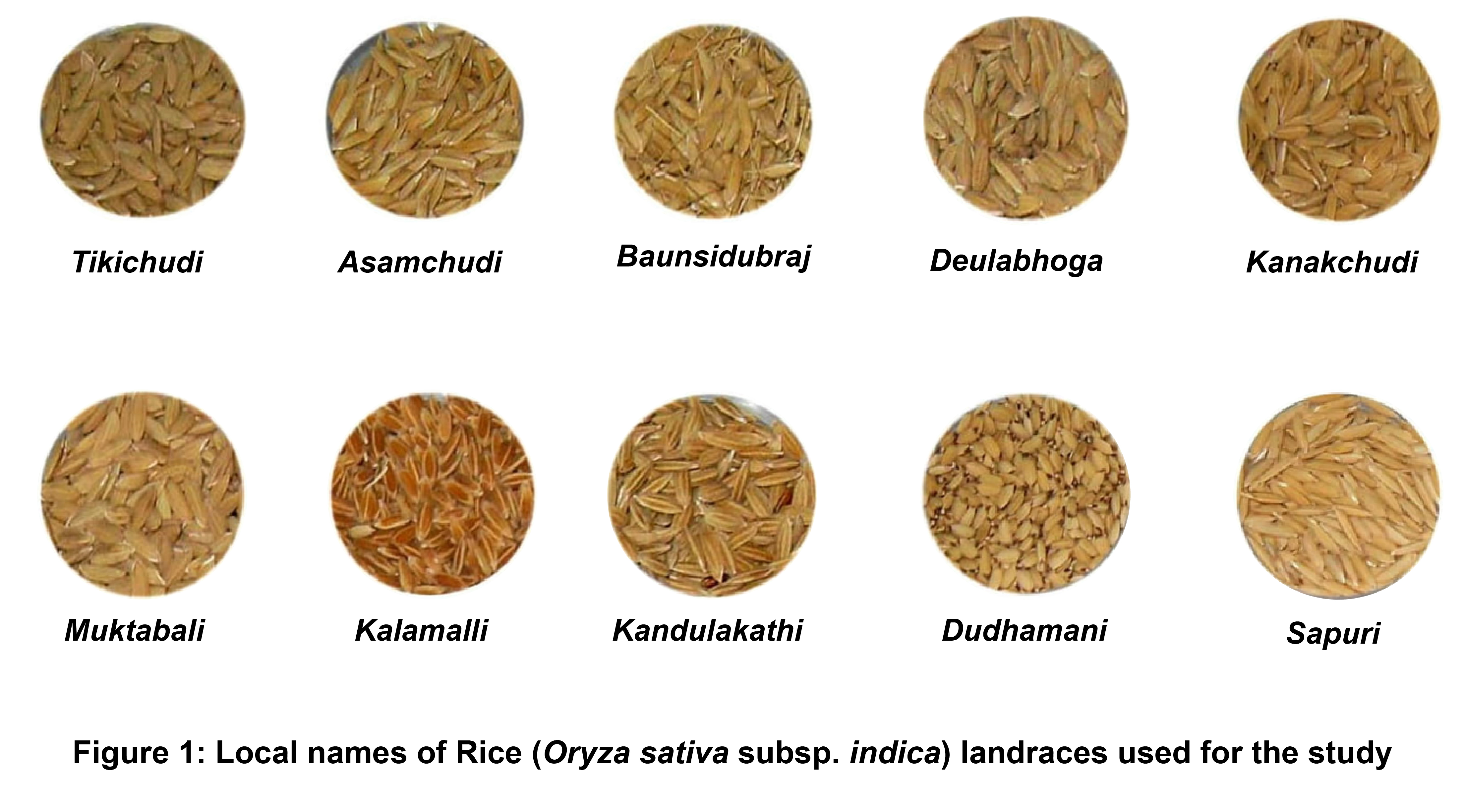

Supplement: Supplementary file 1 — Figure S1 [file FSN3-12-227-s009.jpg]

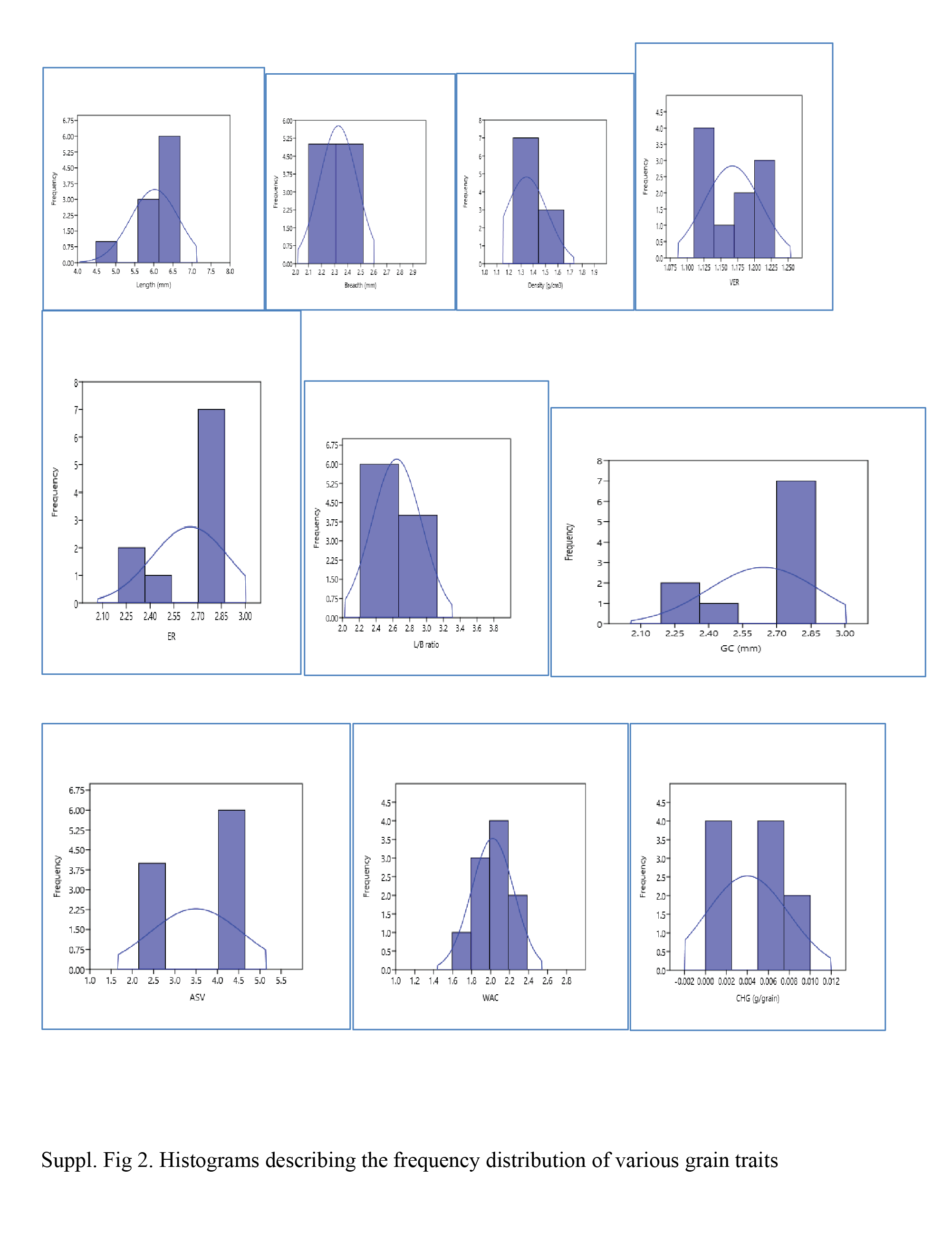

Supplement: Supplementary file 2 — Figure S2 [file FSN3-12-227-s002.png]

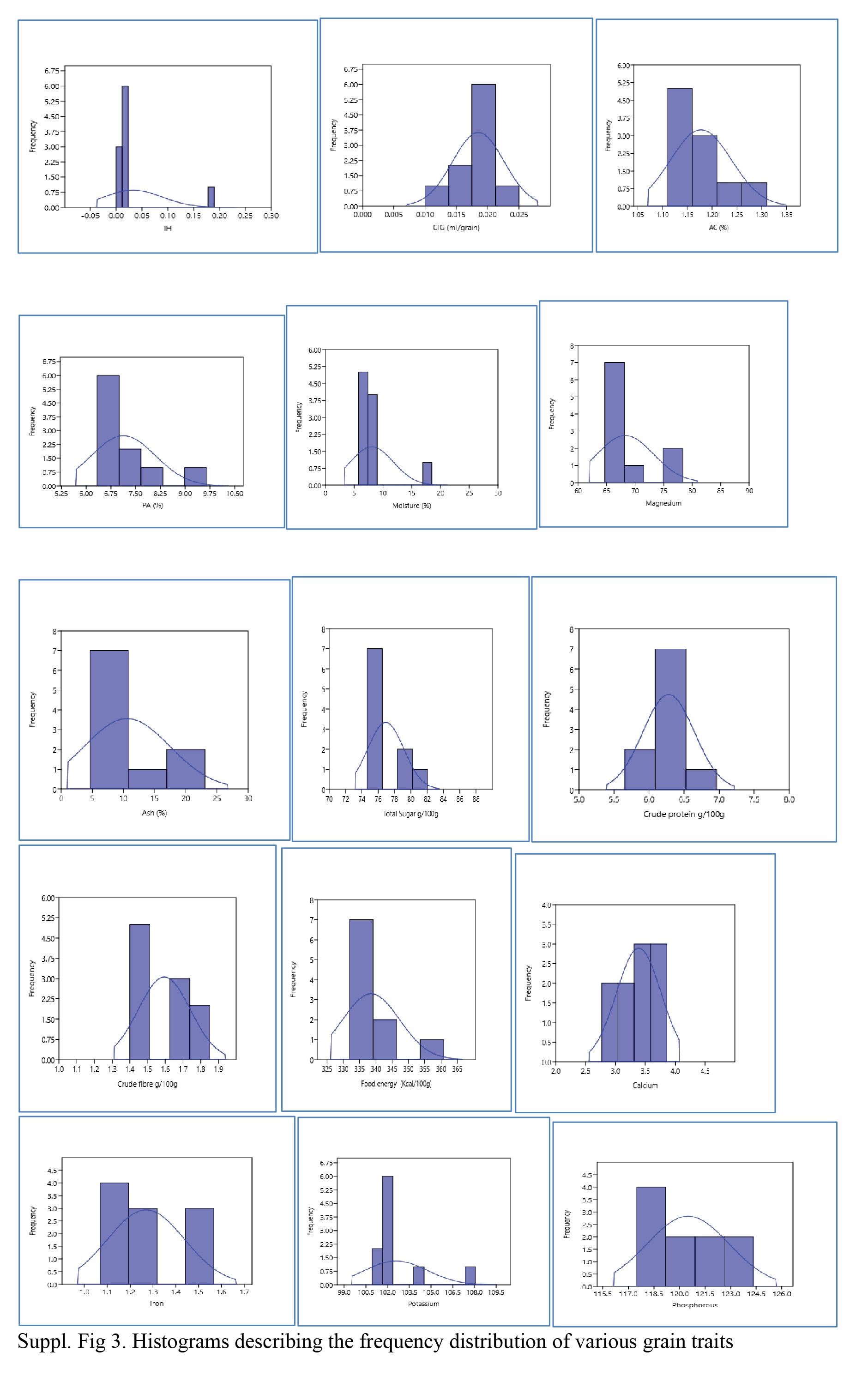

Supplement: Supplementary file 3 — Figure S3 [file FSN3-12-227-s007.png]
